# Simultaneous quantification of human herpesvirus 8 DNA by real time PCR in different tissues of HIV infected cuban patients with Kaposi's sarcoma

**DOI:** 10.1186/2042-4280-1-3

**Published:** 2010-12-07

**Authors:** Vivian Kourí, Pedro A Martínez, Orestes Blanco, Virginia Capó, María E Rodríguez, María del C Dovigny, Lidia Cardellá, Angela Gala, Narciso A Jiménez, Consuelo Correa, Yoan Alemán, Lissette Pérez, Alina Álvarez, Ulrich Hengge

**Affiliations:** 1 Institute of Tropical Medicine "Pedro Kourí", Havana City, Cuba; 2 Latin-American School of medicine, Havana City, Cuba; 3 Skin Center, Düsseldorf, Germany

## Abstract

In Cuba, previous reports have shown an increase of epidemic KS, reaching a total of 120 cases by the end of 2007, despite the use of HAART. To evaluate and compare the role of human herpes virus 8 (HHV-8) viral loads in different compartments of AIDS-related Kaposi's sarcoma (AIDS-KS) patients real-time polymerase chain reaction (RT-PCR) was used to determine the genome copy number of HHV-8 in plasma, saliva, tissue and peripheral blood mononuclear cells (PBMC) of 49 AIDS-KS patients. Overall, 98% of AIDS-KS patients harbored detectable HHV-8. HHV-8 could be detected in 91.6% of KS tissue lesions showing the highest viral load (median log = 3.14 copies/100 ng DNA) followed by saliva and PBMC which were positive in 78%, and 69.2%; respectively. In contrast, HHV-8 was detected in only 37% of plasma samples, which also showed lower viral loads. Men who had sex with men (MSM) were more likely to have three-times higher HHV-8 genome copies in KS lesions when compared with tissues from heterosexuals individuals (OR 3; 95% CI 1.1 to 12.5). These results emphasize the systemic nature of HHV-8-infection and demonstrate the possible role of saliva in HHV-8 transmission among MSM.

## Findings

One hundred and forty-two clinical samples belonging to 49 patients with epidemic KS histologically diagnosed in the Pathology Department at the Institute of Tropical Medicine Pedro Kourí between 2004-2007 were included. The research was approved by local and national ethics committees; all subjects provided their written informed consent. Clinical, immunological and epidemiological data from each patient are depicted in Table 1.

**Table 1:** Demographical, epidemiological and clinical characteristic of the study population

Demographical, epidemiological and clinical variables	AIDS-KS N = 49	
Mean Age	38.2 years (Range: 22-57)	

Gender	Female	2 (4.1%)
	
	Male	47 (95.9%)

Race	White	35 (71.4%)
	
	Mulatto	9 (18.4%)
	
	Black	5 (10.2%)

Sexual orientation	Heterosexual	5 (10.2%)
	
	Homosexual	44 (89.8%)

Type of KS according to macroscopic classification	Cutaneous	35 (71.4%)
	
	Mucocutaneous	7 (14.3%)
	
	Disseminated	7 (14.3%)

Histological classification	Macular	16 (32.6%)
	
	Patch	7 (14.3%)
	
	Tumor	14 (28.6%)
	
	Not classified	12 (24.5%)

Mean HIV viral load* (copies/mL)	65 135 (Range: < 50-580 000)	

Mean T CD4+ cell count (cel/mm^3^)	295 (Range: 8-974)	

T CD4+ cell count	< 200	21 (42.9%)
	
	
	200-499	21 (42.9%)
	
	> 500	7 (14.2%)

Mean number of years after KS diagnosis	1.6 years	

*Data not available from 7 patients.

Different types of samples (41 saliva, 48 tissues, 26 PBMC, and 27 plasma) obtained from each individual were tested at the same time. PBMC and plasma were obtained by separation of 20 mL of citrated whole blood using a Ficoll separation gradient (SIGMA, UK). Paraffin was removed from tissues by xylene treatment according to published protocols [1]. DNA extraction was performed using the QIAamp^® ^DNA Mini Kit (QIAGEN, Germany) and the genomic DNA (gDNA) concentration was determined using spectrophotometer (GeneQuant II, Pharmacia Biotech, EUA) and adjusted to 100 ng, with the exception of plasma where 10 uL were directly used since it was not possible to quantify the gDNA. In order to obtain the standard DNA for absolute quantification, gDNA was extracted from the BCBL-1 cell line and the DNA copy numbers was determined by spectrophotometer as well. Then, the OD concentration was converted to DNA copy number following methods published elsewhere [2]. Once the gDNA copy number from BCBL-1 cell line was calculated, it was adjusted to 1 million copies and a standard curve with serial ten-fold dilutions (from 10^6 ^to 10 copies) assayed in triplicates was prepared. Once the run finished, the standard curve was automatically generated by the *LightCycler *software version 3.3, using the Second Derivative Maximum (SDMM) method. In additon, the detection of Human β globin gene was used as internal control in each clinical sample (with exception of plasma) for measuring of the exact amount of input DNA [3].

RT-PCR primers and conditions were described previously by Watzinger *et al *[4] with minor modifications adapted for the LightCycler 1.5 [4]. Samples were considered negative if the Ct value exceeded cycle 40, or if the copy number was below 10 copies. All patients or samples with more than 10 copies were considered positive, thus infected with HHV-8. For statistical analysis SPSS 11,5 (Inc. SPSS, Chicago, IL, the USA) and Statgraphic were used. Tests for comparison of proportions between the averages were performed using the *Chi-square *test and the Pearson coefficient of correlation for 95% confidentiality.

To our knowledge, this is the first report, where HHV-8 viral load has been simultaneously determined in four different fluids and cells (affected tissue, saliva, plasma and PBMC) from the same AIDS-KS patients. Forty-eight (98%) of the 49 AIDS-KS patients had detectable levels of HHV-8 DNA at least in one of the samples studied, (67% of patients had more than 2 samples positives), with the virus being more frequently detected in KS lesions (44 tissues, 91.6%) followed by saliva in 78% and PBMC in 69.2%. The detection probability and viral load being significantly lower in plasma (37%). There was a positive correlation between the detection of HHV-8 in tissue and the detection in saliva or PBMC (p < 0.01) that was not observed for plasma (Table 2). Although PCR inhibitions could be a possible explanation for the 4 negative results from KS lesions, several other factors that may have limited KSHV detection, like lesion sampling, since the lesion sample was not always the same for molecular detection and diagnosis. In addition, a possible histological inaccuracy could not be excluded because some patients were diagnosed as early stage of KS [5]. However, the percentage of KSHV DNA detected in the present study is similar to previous results published by Mendez and colleagues [6] and higher than the 87% of KSHV DNA detection reported by Kennedy and co-authors [7].

**Table 2:** KSHV load levels in different fluid and cells from each KS patient.

ID	Tissue copies/100 ng gDNA	PBMC copies/100 ng gDNA	Saliva copies/100 ng gDNA	Plasma copies/uL
ADS-KS1	293 (fresh frozen tissue)	41	71	ndt

ADS-KS2	28200 (fresh frozen tissue)	32	193	ndt

ADS-KS3	17670 (fresh frozen tissue)	22	134	ndt

ADS-KS4	51610 (fresh frozen tissue)	130	62	ndt

ADS-KS5	294 (fresh frozen tissue)	ndt	269	92

ADS-KS6	167 (paraffin embedded)	40	130	ndt

ADS-KS7	15850 (fresh frozen tissue)	ndt	ndt	ndt

ADS-KS8	30000 (fresh frozen tissue)	ndt	145	ndt

ADS-KS9	ndt (paraffin embedded)	ndt	ndt	ndt

ADS-KS10	78 (paraffin embedded)	119	121	239

ADS-KS11	1952 (fresh frozen tissue)	38	191	39

ADS-KS12	21840 (fresh frozen tissue)	390	96	49

ADS-KS13	529 (paraffin embedded)	ndt	ndt	2452

ADS-KS14	37630 (fresh frozen tissue)	ndt	394	ndt

ADS-KS15	26090 (fresh frozen tissue)	19	ndt	ndt

ADS-KS16	57 (paraffin embedded)	ndt	77	ndt

ADS-KS17	23 (paraffin embedded)	ndt	49	68

ADS-KS18	477 (paraffin embedded)	307	23	30

ADS-KS19	42 (paraffin embedded)	31	160	ndt

ADS-KS20	1725 (fresh frozen tissue)	ndt	145	ndt

ADS-KS21	ndt (fresh frozen tissue)	42	2482	ndt

ADS-KS22	0 (fresh frozen tissue)	40	584	ndt

ADS-KS23	22 (paraffin embedded)	26	343	20

ADS-KS24	1418000 (fresh frozen tissue)	19	236	65

ADS-KS25	36460 (fresh frozen tissue)	167	32	55

ADS-KS26	749 (fresh frozen tissue)	NS	172	NS

ADS-KS27	47050 (fresh frozen tissue)	NS	NS	NS

ADS-KS28	58 (fresh frozen tissue)	NS	ndt	NS

ADS-KS29	389 (fresh frozen tissue)	NS	ndt	NS

ADS-KS30	39830 (fresh frozen tissue)	NS	89	NS

ADS-KS31	10040 (paraffin embedded)	NS	NS	NS

ADS-KS32	819 (fresh frozen tissue)	NS	NS	NS

ADS-KS33	128800 (fresh frozen tissue)	NS	445	NS

ADS-KS34	ndt (fresh frozen tissue)	NS	366	NS

ADS-KS35	206 (fresh frozen tissue)	NS	ndt	NS

ADS-KS36	68280	NS	114	ndt

ADS-KS37	877 (paraffin embedded)	NS	NS	NS

ADS-KS38	30730 (fresh frozen tissue)	NS	131	NS

ADS-KS39	NS	53	9447	ndt

ADS-KS40	867 (paraffin embedded)	NS	ndt	NS

ADS-KS41	30520 (fresh frozen tissue)	NS	148	NS

ADS-KS42	35 (fresh frozen tissue)	NS	NT	NS

ADS-KS43	31740 (fresh frozen tissue)	NS	177	NS

ADS-KS44	10120 (fresh frozen tissue)	NS	132	NS

ADS-KS45	139800 (fresh frozen tissue)	NS	NS	NS

ADS-KS46	2483 (fresh frozen tissue)	NS	ndt	NS

ADS-KS47	163 (fresh frozen tissue)	NS	NS	NS

ADS-KS48	28680 (fresh frozen tissue)	NS	230	NS

ADS-KS49	58 (paraffin embedded)	NS	NS	NS

**Abbreviations: Ndt: **non detectable**; NS: **no sample available.

All fresh frozen tissues, PBMC and saliva samples, were confirmed to have the same amount of the input gDNA in100 ng, by detecting similar crossing point (Cp) amplification signal of the Human β globin gene among them (average Cp: 24.08, range: 22-25); in contrast, paraffin-embedded tissues showed a gDNA amplification signal after cicle 29 (range:27-32), probably due to degradation [8]. This result was consistent with the detection of HHV-8 load significantly higher in fresh frozen tissue than in paraffin-embedded tissue (p < 0.003) (Table 2).

**Figure 1:**
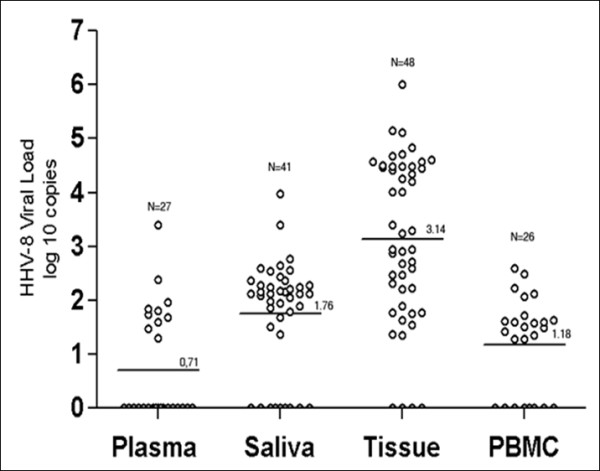
**Comparison of the logarithmic KSHV load detected in different samples of epidemic KS patient using the RT-PCR assay of a conserved region of ORF-26**.

Despite the difference detected between fresh frozen and paraffin-embedded tissue, when copy numbers from different samples (tissue-plasma, tissue-saliva, tissue-PBMC, plasma-saliva, PMBC-saliva, and PBMC-plasma) within a given individual were compared, a significantly higher HHV-8 viral load in KS lesions compared to all the other samples (p < 0.05) was detected (Figure 1). One of the reasons that may explain why the higher HHV-8 load is detected in tissue, specifically in the case of HIV infection, is that the HIV *tat *protein promotes the replication of HHV-8-stimulated cell proliferation and inhibits apoptosis of spindle cells infected with HHV-8 [9,10]. Recent reports have described a higher HHV-8 load of AIDS-KS lesions compared with endemic KS, although the differences were not statistically significant.

HHV-8 load in saliva and PBMC was significantly lower than in tissue (p < 0.05) and significantly higher than in plasma (p < 0.05; Figure 1), however there were no statistic differences between the viral load in saliva and PBMC (p > 0.05). Of note, saliva represented the second most frequent source for HHV-8 detection (Figure 1). In the light of previous reports [11-13] and consistent with the present results transmission by saliva may contribute to the spread of HHV-8 infection among the HIV seropositive population besides sexual intercourse [14]. It still remains controversial why, if saliva is the main source of virus, HHV-8 infection shows a sexual pattern of transmission. Nevertheless, saliva is the only mucosal fluid in which infectious HHV-8 has been identified, although factors associated with HHV-8 salivary shedding remain unclear [15]. Moreover, future research will need to assess the possible impact of saliva in HHV-8 transmission among Cuban HIV seronegative individuals.

Most authors agree that the viral genome present in PBMC of AIDS-KS patients occurs in a latent state, but the role of HHV-8 persistence in PBMC with regard to the pathogenesis of KS remains unclear [16]. The lower copy number of HHV-8 detected in PBMC in comparison to KS tissue, could be due to its latent state within this compartment, in contrast to viral expression present in lesional spindle cells [17].

Harrington and colleagues demonstrated that the presence of HHV-8 in plasma is intermittent [18]. For other viral infections, plasma viral load has been used as the best marker to estimate disease progression [19-21]. However, this does not seem to be true for KS and HHV-8, as has been proposed by Polstra and colleagues [22]. Hundred percent of Cuban AIDS-KS patients, in whom HHV-8 was detected in plasma also tested positive in other clinical samples (saliva, KS tissue, PBMC). Thus, viremia could occur in those patients with active viral replication, alternating with latency periods where the virus is not detected in plasma, as has been reported for other members of the *Herpesviridae *family [23].

**Figure 2:**
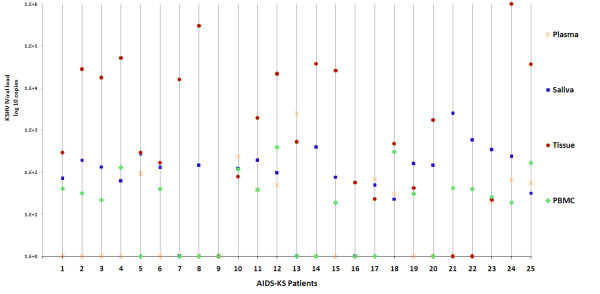
**Graphic representation of KSHV loads in different samples per each AIDS-KS patient**.

There was a significant Pearson's correlation when each patient's tissue HHV-8 levels were compared with saliva and PBMC (p < 0.01); however, this correlation did not exist for plasma samples (Figure 2). We did not find association between the HHV-8 viral load in all the samples analyzed with: CD4 cell counts, HIV viral load, type of KS (cutaneous, mucocutaneous and systemic), the histological stage (macular, patch and tumoral) nor the number of years elapsed between the KS diagnosis and death (p > 0.05).

In our study, Caucasian AIDS-KS patients were more likely to be positive for HHV-8 infection than black or mulatto KS patients (p = 0.006). There is no previous report regarding the association of HHV-8 levels with skin color. However, it has been described several times that AIDS-KS is more frequent in individuals with white skin [24]. Some authors have proposed a genetic predisposition to HHV-8 infection in individuals with certain HLA types (A*6801 and DRB1*04) that tend to have increased viral excretion in saliva e.g. in African women [25]. MSM were more likely to have three-times higher HHV-8 genome copies in KS lesions when compared with tissues from heterosexuals individuals (OR 3; 95% CI 1.1 to 12.5). This seems to be a very important finding that confirms what has been reported by other authors regarding sexual risk behaviors that are more prevalent in homosexual intercourse [26]. With the present study we confirm that HHV-8 produces a systemic infection in different body compartments and that the highest HHV-8 levels were produced in lesional KS tissue. Furthermore, saliva has been recognized as an important reservoir for HHV-8 transmission.

One of the main challenges for researchers is to identify the exact mode of virus transmission following sexual intercourse, as the presence of HHV-8 in semen, anal secretions or vaginal fluid has not been consistently demonstrated [27-30].

## Competing interests

The authors declare that they have no competing interests.

## Authors' contributions

All authors read and approved the final manuscript. VK and PAM participated in the conception and design, acquisition of data, analysis and interpretation of data, drafted the manuscript. OB, MER, MdCD and NAJ made the clinical diagnosis and patients follow up, sampling, clinical analysis and interpretation of data. LC and AG participated in the design of the study and performed the statistical analysis. CC, YA, LP and AÁ carried out the molecular genetic studies. Finally, UH participated in the design of the study and drafted the manuscript.
